# Dynamics of maternally transferred trace elements in oyster larvae and latent growth effects

**DOI:** 10.1038/s41598-017-03753-2

**Published:** 2017-06-15

**Authors:** Nanyan Weng, Wen-Xiong Wang

**Affiliations:** 1 Marine Environmental Laboratory, HKUST Shenzhen Research Institute, Shenzhen, 518057 China; 2 Center for Marine Environmental Chemistry and Toxicology (CMECT), College of Environment and Ecology, Xiamen University, Xiamen, 361005 China

## Abstract

Understanding the maternal transfer of contaminants and their potential effects has great implications for a valid ecological assessment of environmental pollution. However, relevant studies on marine bivalves are very limited. Here, we examined the maternal transfer of trace metals in populations of oyster *Crassostrea hongkongensis* with contrasting metal exposure histories. Elevated accumulation of trace metals was observed in eggs and larvae from contaminated sites, suggesting maternal transfer of multi-metals in natural oyster populations. The dynamics of maternally transferred metals was for the first time documented in this study. We demonstrated that excessively transferred metals in contaminated larvae were rapidly eliminated during the early developmental stage, and the efflux rate of metals in larvae was greatly dependent on environmental contamination level. These results provided the first field evidence of modified metal biokinetics in offsprings due to exposure history of adults in marine bivalves. Moreover, egg production was negatively correlated with the contamination level of metals in eggs. There was a further lagged growth in the contaminated larvae, indicating the potential adverse and latent effects of maternally transferred metals on the viability of oyster offspring. Our findings highlighted the importance of transgenerational studies on long-term metal exposure in marine bivalves.

## Introduction

Maternal transfer of contaminants has attracted increasing attention because contaminants in adult organisms may be maternally transferred and result in a series of influences on their offsprings. Reduced success of reproduction and survival of offspring caused by maternal transferred contaminants has been demonstrated in many aquatic organisms^1–3^. Among various kinds of environmental contaminants, trace metal pollution has been considered as one of the most important threats to the coastal and estuarine organisms in China^4, 5^. Filter feeders such as oysters are the major sedentary species in estuarine environments, and play an important role in community structure and maintenance of the ecosystem^6^. Compared to other organisms, oysters have strong ability to accumulate trace metals, especially in contaminated environments. Recently, blue (*Crassostrea hongkongensis*) and green (*Crassostrea sikamea*) colored oysters with tremendous metal accumulation have been found in estuaries of Southern China^7–9^. High accumulation of metals in gonad tissue of oysters from contaminated sites has also been observed. For instance, the concentrations of Cu, Cr, Ag and Cd in the gonad of contaminated oysters (*Crassostrea hongkongensis*) were up to 21.9-, 17.9-, 7.2-, and 3.8-fold higher than those of the clean oysters^10^. Such high level of trace metals in gonad tissue of oysters may result in the exposure of early life stages (i.e. gametes, embryos and larvae) that are very sensitive to contaminants^11^. Therefore, it is important to understand whether large amounts of metals accumulated in the contaminated oysters can be maternally transferred and subsequently exert an influence on their offspring. Indeed, the population numbers of shellfish in several contaminated estuaries of Southern China have been greatly decreased in recent years, and it is relevant to study whether maternal transfer of metals contributed to such declines.

Maternal transfer of trace elements has been widely reported in amphibians^12–14^, fish^3, 15^, birds^16, 17^ and mammals^18^, but the available data on marine invertebrates including bivalves are limited, especially for those seriously contaminated populations. Earlier, one laboratory exposure study examined Hg transfer in mussels *M*. *edulis* ^19^, while the other two field studies focused on the oysters of *Crassostrea virginica* ^20^ and *Crassostrea sikamea* ^21^. Only a few metals (Cu, Zn and Cd) were considered in these two studies and the potential biological effects have not yet been revealed. Moreover, most previous studies were limited to the observation of contaminants in maternal tissues and eggs of females, whereas offspring were rarely considered. Only a few studies determined the levels of transferred metals in the new hatchlings of Japanese medaka (*Oryzias latipes*)^22^ and Pacific Leatherback turtles (*Dermochelys coriacea*)^23, 24^. The dynamics of maternal transferred contaminants in early life stages of offspring has been not investigated until now.

In the present study, we sampled oyster (*Crassostrea hongkongensis*) populations with contrasting metal exposure histories from several estuaries of Southern China. We then investigated whether oyster offspring from contaminated sites presented higher levels of trace element than those of the relatively clean sites as an indication of maternal transfer, and the dynamics of the maternal transferred metals in the early developmental stage of oyster larvae. The variations between elements and populations were also examined. Moreover, egg production, egg size as well as larvae growth of different oyster populations were determined to explore the potential effects of maternal transfer of trace elements. This study provided further understanding of the impacts of long-term metal pollution on marine bivalves.

## Results and Discussions

### Trace metal concentration in females, eggs and larvae

In this study, concentrations of 10 trace elements (Ag, As, Cd, Co, Cr, Cu, Ni, Pb, Se, and Zn) in females, eggs and newly hatched larvae (24 h post-fertilization) of oyster *Crassostrea hongkongensis* from several estuaries were determined. Metal concentrations in females varied greatly among different oyster populations, which provided an excellent system to investigate the maternal transfer of trace metals in the field. Females collected from the contaminated sites (BJ, STP and SZ) had significantly higher body concentrations of trace elements (*p* < 0.05, Tukey test) compared with females from the relatively clean sites (STR and JZ). Specifically, the ratios between the maximum and minimum concentrations of Cu, Zn, Cr, Ag, Co and Cd in females from all sites were up to 26.6, 6.4, 11.5, 13.4, 19.9 and 6.3, respectively (Table 1). The concentrations of trace metals measured in the oysters of this study were consistent with the previous reports of oysters in the corresponding estuaries^7–9^.

**Table 1:** Trace element concentrations in female oysters, eggs and new hatched larvae (24 h post-fertilization) from each site. Values are given as mean ± SD.

	Cu	Zn	Cr	Ni	Co	Cd	Ag	Pb	Se	As
Site	Females (µg/g, dry weight)*									
BJ	10916 ± 2756c	17123 ± 1764b	4.50 ± 0.92c	5.42 ± 1.01b	2.82 ± 1.06c	17.6 ± 4.32c	7.52 ± 3.35d	1.49 ± 0.24b	5.75 ± 0.30b	19.2 ± 1.90e
SZ	4328 ± 1252b	11481 ± 3378a	0.75 ± 0.24b	6.03 ± 1.71b	0.65 ± 0.25b	22.7 ± 2.83cd	3.57 ± 1.35c	1.40 ± 0.28b	7.08 ± 0.58c	6.85 ± 0.67c
JZ	499 ± 140a	8372 ± 1873a	0.39 ± 0.10a	2.03 ± 0.50a	0.29 ± 0.06a	4.27 ± 0.77a	0.56 ± 0.24ab	0.84 ± 0.19a	6.17 ± 0.62b	8.62 ± 1.74d
STR	410 ± 129a	10323 ± 2153a	0.47 ± 0.13a	2.49 ± 0.46a	0.62 ± 0.23b	12.8 ± 3.30b	0.84 ± 0.26a	0.82 ± 0.13a	4.86 ± 0.52a	5.02 ± 0.83b
STP	3037 ± 1009b	56292 ± 11476c	0.80 ± 0.17b	8.08 ± 3.30c	5.78 ± 1.68d	27.0 ± 5.78d	1.53 ± 0.72b	0.86 ± 0.15a	5.53 ± 0.67b	3.68 ± 0.54a
Eggs (µg/g, dry weight)*										
BJ	642 ± 127c	1098 ± 149c	13.3 ± 5.41c	5.16 ± 1.51b	0.91 ± 0.39c	0.86 ± 0.34b	1.31 ± 0.74c	1.46 ± 0.98c	4.51 ± 1.83b	9.42 ± 5.34a
SZ	264 ± 83.4b	486 ± 102b	9.03 ± 3.30b	9.86 ± 3.23c	0.27 ± 0.09b	0.78 ± 0.19b	0.43 ± 0.12b	0.93 ± 0.27c	6.17 ± 1.92b	16.2 ± 6.39b
JZ	45.3 ± 21.0a	300 ± 87.8a	0.71 ± 0.39a	1.42 ± 1.03a	0.07 ± 0.03a	0.21 ± 0.11a	0.07 ± 0.03a	0.48 ± 0.20b	4.76 ± 1.16b	6.11 ± 1.08a
STR	51.0 ± 14.2a	473 ± 106b	7.95 ± 3.22b	4.88 ± 1.94b	0.37 ± 0.20b	0.95 ± 0.42b	0.10 ± 0.03a	0.73 ± 0.28bc	2.58 ± 0.83a	5.79 ± 1.68a
STP	249 ± 30.2b	3456 ± 704d	7.27 ± 3.48b	10.6 ± 3.82c	3.13 ± 1.03d	1.80 ± 0.31c	0.33 ± 0.14b	0.21 ± 0.04a	4.46 ± 2.55ab	4.26 ± 0.83a
Larvae (µg/g, dry weight)**										
BJ	108 ± 9.31c	177 ± 17.5c	3.33 ± 0.17c	2.64 ± 0.26bc	0.36 ± 0.03c	0.29 ± 0.03b	0.31 ± 0.05c	0.55 ± 0.01b	3.27 ± 0.29b	8.12 ± 0.93ab
SZ	55.4 ± 2.55b	88.5 ± 7.67b	2.22 ± 0.02b	3.84 ± 0.29b	0.11 ± 0.02b	0.40 ± 0.09b	0.13 ± 0.01b	0.62 ± 0.10b	4.04 ± 0.90b	16.5 ± 1.27b
JZ	9.81 ± 0.44a	39.9 ± 4.21a	0.88 ± 0.22a	0.64 ± 0.16a	0.05 ± 0.00a	0.06 ± 0.01a	0.04 ± 0.01a	0.19 ± 0.04a	1.82 ± 0.10ab	4.46 ± 0.35a
STR	13.5 ± 2.7811a	70.8 ± 5.07b	1.03 ± 0.21a	1.24 ± 0.47ab	0.10 ± 0.02b	0.25 ± 0.06b	0.05 ± 0.01a	0.31 ± 0.05a	1.30 ± 0.43a	4.32 ± 1.55a

Different letters in the same column indicated significant difference among sites at *p* < 0.05 level (one-way ANOVA, Tukey test), “*”n = 8 for BJ, SZ and STR site and n = 7 for JZ and STR site; “**”two independent replicates for each site, about 100,000–120,000 larvae from at least 10 females and 4 males for each replicate.

Comparing among sites, higher concentrations of most of the determined elements were also observed in eggs and larvae from contaminated sites as compared to those of the relatively clean sites. For example, Cu, Zn, Cr and Ni concentrations in larvae from contaminated BJ site were enriched by 11.1, 4.4, 3.8 and 3.9 times relative to the metal levels in the relatively clean JZ larvae, respectively (Table 1). Similar pattern was also presented in oyster eggs. Moreover, the concentrations of trace elements (Cu, Zn, Cr, Ni, Cd, Co, Ag, Se) in newly hatched larvae were significantly and positively related to the concentrations in females (Spearman rank correlation, *p* < 0.05, Figure S1), suggesting the transfer of these elements from exposed adults to offspring via gametes. Maternal transfer of Cu and Zn were also observed in field populations of *Crassostrea sikamea* ^21^. However, Grieg *et al*.^20^ found that *Crassostrea virginica* from polluted areas with high tissue concentrations of Cu and Cd produced eggs with comparable metal contents to those produced by oysters from the reference site. This may have been due to the slight difference of metal contamination levels between two sites. Even though one previous study suggested that gonad tissue was not the main storage organ of trace metals in bivalves^25^, our results provided clear evidence of the maternal transfer of multi-metals in marine bivalves. Bivalves such as oyster *Crassostrea hongkongensis* with strong ability to accumulate trace metals may implicate a great possibility of maternal transfer, especially in the seriously contaminated environments.

Moreover, there were also obvious differences of maternal transfer among elements. The concentrations of Cu, Zn, Cd and Ag in eggs and larvae were substantially lower than those in maternal female tissues (Table 1), suggesting a great capacity of regulating the metabolism of these metals in oysters to avoid their accumulation in oocyte. For instance, Cu and Ag concentrations in the somatic tissues of females were 8.0–17.0 folds and 4.6–8.4 folds higher than those of their corresponding eggs, respectively. Given the high reproductive output of oysters, reproduction could be a possible way for trace element elimination in oysters. The egg burdens of Cu, Zn, Cd and Ag accounted for only a small proportion (<3%, Table S2) of the total body burden of the corresponding metals in the females of all populations. Thus, the potential contribution of spawning on the depuration of these metals in oysters was negligible. The restricted transfer of Cd and Ag from female to eggs was also observed in reptiles^26^, birds^17^, and cladocerans^27^. Nevertheless, Metts *et al*.^14^ demonstrated the high transfer potentials of Cu and Zn in *Bufo Anaxyrus terrestris*, and the egg concentrations of these elements were 1.1 and 1.2 times higher than the corresponding element in females, respectively. Thus, maternal transfer of metals also varied greatly among different species.

In contrast, Cr, Ni, Se and As were more inclined to accumulate in the oyster eggs, especially in the contaminated sites. For instance, Cr concentrations in the relatively clean JZ site were comparable between eggs and somatic tissues, while its accumulation in eggs was at least 3-fold higher than that in somatic tissues of females (Table 1) in the contaminated sites (BJ, SZ and STP). The highest percentages of Cr, Ni, As and Se deposited in eggs were up to 77.4%, 28.4%, 28.6% and 16.4%, respectively (Table S2). Consequently, as a route for elimination in oysters, maternal transfer was especially important for these elements. Chen *et al*.^22^ found that approximately 61% of Cr accumulation in Japanese medaka (*Oryzias latipes*) was eliminated by spawning. Similarly, Se (35%) was maternally transferred to offspring in *Daphnia magna* ^27^. High maternal transfer efficiency of Se was also observed in several amphibian species such as *Gastrophryne carolinensis* (53%)^28^ and *Sceloporus occidentalis* (33%)^29^.

### Dynamics of maternally transferred trace elements in larvae

**Figure 1:**
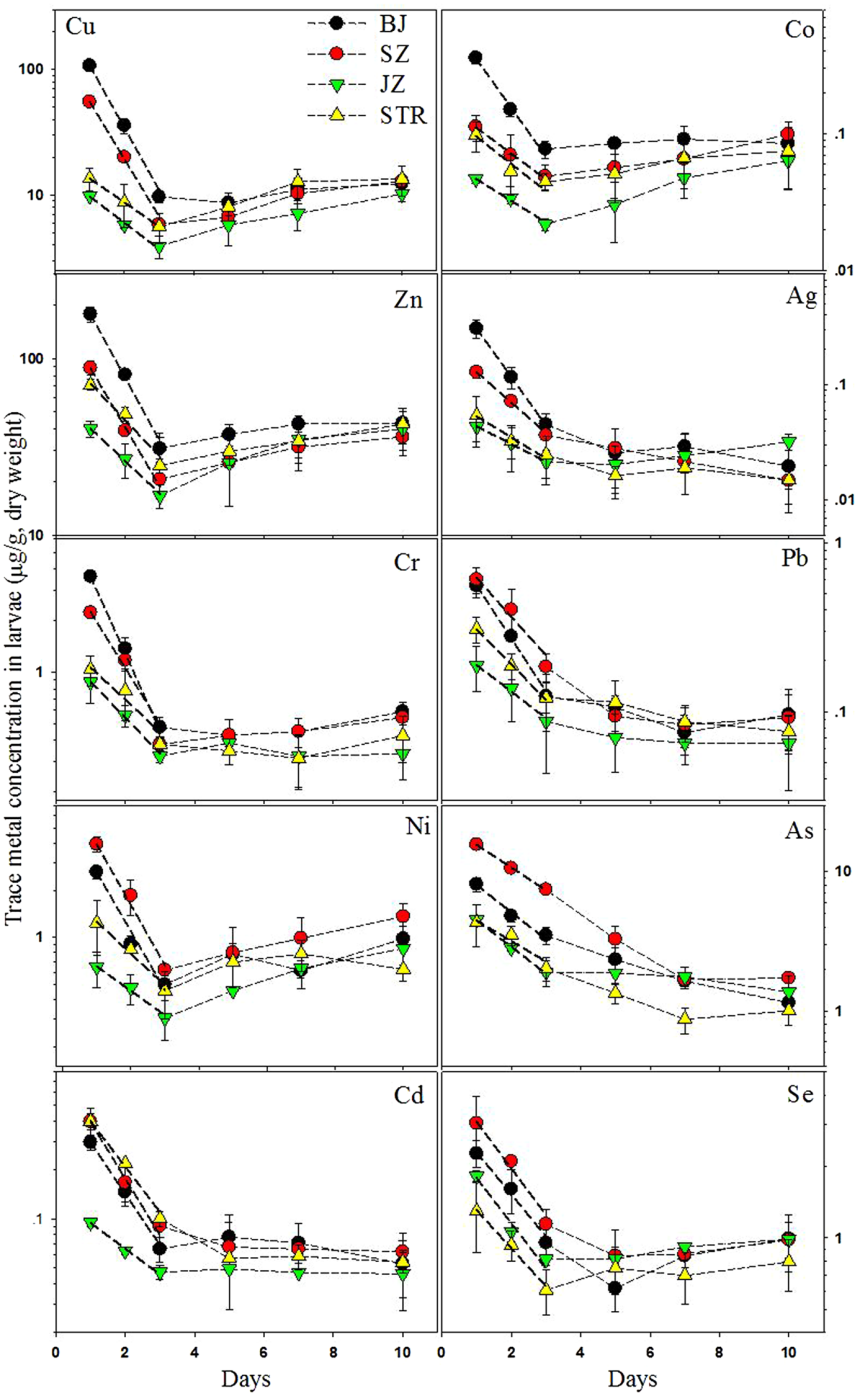
Temporal changes of trace element concentrations in oyster larvae from each site during the early development stage. The bold dotted line shows the fitting curve of the trace element dynamics for the first two days modeled by equation 1. Values are given as mean ± SD, two independent replicates for each site, and larvae from at least 10 females and 4 males for each replicate (the number of larvae is about 100,000–120,000 for each replicate at each time point).

The temporal dynamics of trace elements in the early development stage of oysters is shown in Figure 1. The concentrations of trace elements in oyster larvae decreased sharply during the first two days, with the reduction by up to 80% for most of the determined elements, and then the concentrations began to level off from the fifth day. Growth dilution effects may greatly attribute to the dramatic decrease of trace element concentration in oyster larvae, since their growth rates were up to 0.33–0.34 d^−1^ (Table 2) during the first two days_._ Interestingly, the discrepancy of metal concentrations among sites was greatly narrowed with time (Figure 1) and there was no significant difference of growth rate among sites, suggesting that the elimination process was involved in such decrease. The dynamics of metal concentrations within the first two days could be well modeled by one compartment model (equations 1 and 2, Figure 1). The efflux rate constants (*k*_*e*_) was thus calculated as the slope of nature log transformed larvae metal concentrations against time subtracting the growth rate (Table 2).

**Table 2:** The efflux rate constant (*k*_e_, d^−1^) of trace elements as well as growth rate (g, d^−1^) of oyster larvae originated from each site during the first two days.

Site	BJ		SZ		JZ		STR	
Element	*k*_e_	*r* ^*2*^	*k*_e_	*r* ^*2*^	*k*_e_	*r* ^*2*^	*k*_e_	*r* ^*2*^
Cu	^C^0.81 ± 0.07d	0.97	^B^0.67 ± 0.05d	0.98	^A^0.12 ± 0.02b	0.95	^A^0.14 ± 0.04b	0.98
Zn	^B^0.50 ± 0.05b	0.96	^B^0.44 ± 0.05c	0.94	^A^0.11 ± 0.02b	0.93	^A^0.15 ± 0.08b	0.99
Cr	^C^0.70 ± 0.15c	0.98	^B^0.42 ± 0.13c	0.87	^A^0.10 ± 0.03b	0.86	^A^0.15 ± 0.06b	0.94
Ni	^C^0.49 ± 0.08b	0.97	^C^0.53 ± 0.04c	0.97	^A^0.02 ± 0.04a	0.97	^B^0.13 ± 0.06b	0.97
Co	^B^0.49 ± 0.06b	0.94	^A^0.10 ± 0.03a	0.85	^A^0.03 ± 0.03a	0.97	^A^0.09 ± 0.05a	0.88
Cd	^C^0.40 ± 0.03b	0.94	^C^0.47 ± 0.07c	0.97	^A^0.03 ± 0.02a	0.90	^B^0.30 ± 0.05c	0.96
Ag	^C^0.67 ± 0.01c	0.92	^B^0.27 ± 0.03b	0.98	^A^0.02 ± 0.01a	0.95	^A^0.08 ± 0.04a	0.97
Pb	^C^0.39 ± 0.05b	0.95	^B^0.20 ± 0.08b	0.96	^A^0.05 ± 0.03a	0.95	^B^0.14 ± 0.05b	0.91
Se	^A^0.07 ± 0.05a	0.83	^A^0.09 ± 0.05a	0.91	^A^0.05 ± 0.02a	0.97	^A^0.05 ± 0.03a	0.94
As	^A^0.09 ± 0.04a	0.98	^A^0.07 ± 0.02a	0.93	^A^0.06 ± 0.04a	0.85	^A^0.05 ± 0.03a	0.96
g	^A^0.33 ± 0.02		^A^0.33 ± 0.03		^A^0.34 ± 0.03		^A^0.34 ± 0.02	

Values are given as mean ± SD (two independent replicates for each site, about 100,000–120,000 larvae from at least 10 females and 4 males for each replicate). Different letters indicate significant difference among elements and sites at *p* < 0.05 level (one-way ANOVA, Tukey test), lowercase letters are used for different elements and capital letters are used for different sites.

Generally, elimination of metals in oyster larvae was significantly faster than that of the adults, especially for the contaminated metals. Earlier study showed that the efflux rates of 8 metals (Ag, Cd, Co, Cr, Cu, Ni, Pb and Zn) were less than 0.11 d^−1^ in the adult oyster *Crassostrea sikamea* from contaminated site in Jiulong river estuary^30^. Similar results were also observed in other two oyster species (*Crassostrea rivularis* and *Saccostrea glomerata*)^31^. Related data on the biokinetics of trace metals in bivalve larvae are very limited. Only two earlier studies compared the accumulation of Cd between adults and larvae of bivalve *Isognomon californicum*, with the accumulation rates of larvae being 5–10 times greater than adult rates^32, 33^. The relatively higher metabolic rate as well as larger ratio of surface area to volume may facilitate the efflux of trace metals in oyster larvae. The larvae oxygen consumption rate was approximately 10 times greater than the adult rates of *Mytilus edulis* ^34^.

Great variations of efflux rates were also observed among trace elements, but were dependent on the sites. Specifically, the efflux rates of Cu, Zn, Cr, Ni and Ag of oyster larvae from contaminated sites (BJ and SZ) were much higher than those of Pb, As and Se. For the JZ larvae, relatively high efflux rate was only observed for Cu, Zn and Cr (≥0.10 d^−1^), the *k*_*e*_ of Ni, Co, Cd, Ag, Se and As was less than 0.06 d^−1^, suggesting that the decreased concentration of these elements in clean oyster larvae were mainly attributed to the growth dilution. The obvious elimination of essential elements such as Cu (*k*e: 0.11) and Zn (*k*e: 0.12) were also observed in the early developmental stages of oysters from relatively clean site (JZ), suggesting that the requirements of Cu and Zn for oocyte development were higher than those for embryonic development and early larval growth. Thus, there was significant stage-specific difference in the requirement or metabolism of these metals in oysters. For instance, Cu has been proved to play an important role in the attachment of oyster larvae^35^, and our recent study found that soft tissue concentration of Cu was maintained at a very low level in early larvae (11–17 µg/g, dry weight), while the content increased to 142–168 µg/g (dry weight) in the eyespot stage of oysters *Crassostrea hongkongensis* (Weng and Wang, unpublished data). Of the elements investigated, As was of particular interest since its efflux rate was especially low (≤0.09 d^−1^) in larvae from all sites and its concentration decreased continuously with time. Organic arsenobetaine was the dominant form of As in marine invertebrates, and is often used as an osmolyte^36^, which may explain the different handling of As in oyster larvae. More research is required to further understand the biological role of different trace elements in the development of bivalve larvae at different life stages.

More importantly, higher efflux rates of trace elements were observed for all elements in contaminated larvae (BJ and SZ), except As and Se with similar values among sites. For instance, the efflux rates of Cu, Zn, Cr and Ni in contaminated BJ larvae were about 2.6-fold, 1.9-fold, 3.7-fold, 6.7-fold higher than those of the relatively clean JZ larvae, respectively. Most of the maternally transferred metals in larvae from contaminated sites were rapidly eliminated. For example, more than 70% of the maternally transferred metals such as Cu (85%), Zn (71%), Cr (76%) and Ag (80%) in contaminated BJ larvae were eliminated during the first two days. The rapid elimination of metals in oyster larvae from contaminated sites may be an important strategy to cope with metal stress. The efflux of trace metals in oyster larvae was greatly dependent on pollution level in the field. For instance, Cu efflux rate of oyster larvae among sites increased following the same order as that of Cu concentration in females: BJ > SZ > STR, JZ. Similar trends also observed in other metals such as Cr, Cd and Ag (Table 2). Alteration of metal biokinetics has been widely reported in the laboratory pre-exposure metal studies as well as in metal contaminated field populations of adult bivalves^37^. For instance, Shi and Wang^38^ found that pre-exposure of Zn, Ag and Cd to the green mussels *Perna viridis* greatly changed the uptake and assimilation of the corresponding metals from both dissolved and food phases. Lower uptake rates of Hg and Ag were observed in the clam *Macoma balthica* from the contaminated estuary when compared with those of clams from the uncontaminated estuary^39^. However, the metal biokinetics of offspring was not investigated in these earlier studies. Our results demonstrated obvious difference of metal efflux rates of larvae between different oyster populations, providing the first evidence of modified metal biokinetics in offspring due to the exposure history of adults in marine bivalves. Similarly, the multi-generational study on *Daphnia magna* also demonstrated that exposure of adults can greatly change the uptake and elimination of metals in the later generations, but such change disappeared after a few generations^27^. Recently, two field studies on adult oysters of *Crassostrea hongkongensis* ^40^ and *Crassostrea sikamea* ^31^ from the same site of this study also demonstrated the enhanced metal elimination of contaminated oysters (BJ site in this study) in comparison to oysters from the relatively clean site (JZ site in this study). The change of metal biokinetics may greatly contribute to the tolerance development of oyster populations living in contaminated environments. It would be interesting to further explore whether similar changes of metal biokinetics in both adults and their offspring of oysters lead to genetic evolution of pollution resistance in the field. Evidence suggested that the offspring produced by contaminated oyster *Crassostrea sikamea* were more tolerant than those from the clean sites, and metallothionein (MT) content was 6-fold higher in contaminated oyster larvae than that of the reference larvae^21^. Further investigations on metallothioneins in different developmental stages are needed to explore its potential role in biokinetics and metabolism of trace metals in larvae of marine bivalves.

### Effects on reproduction and larval growth

**Figure 2:**
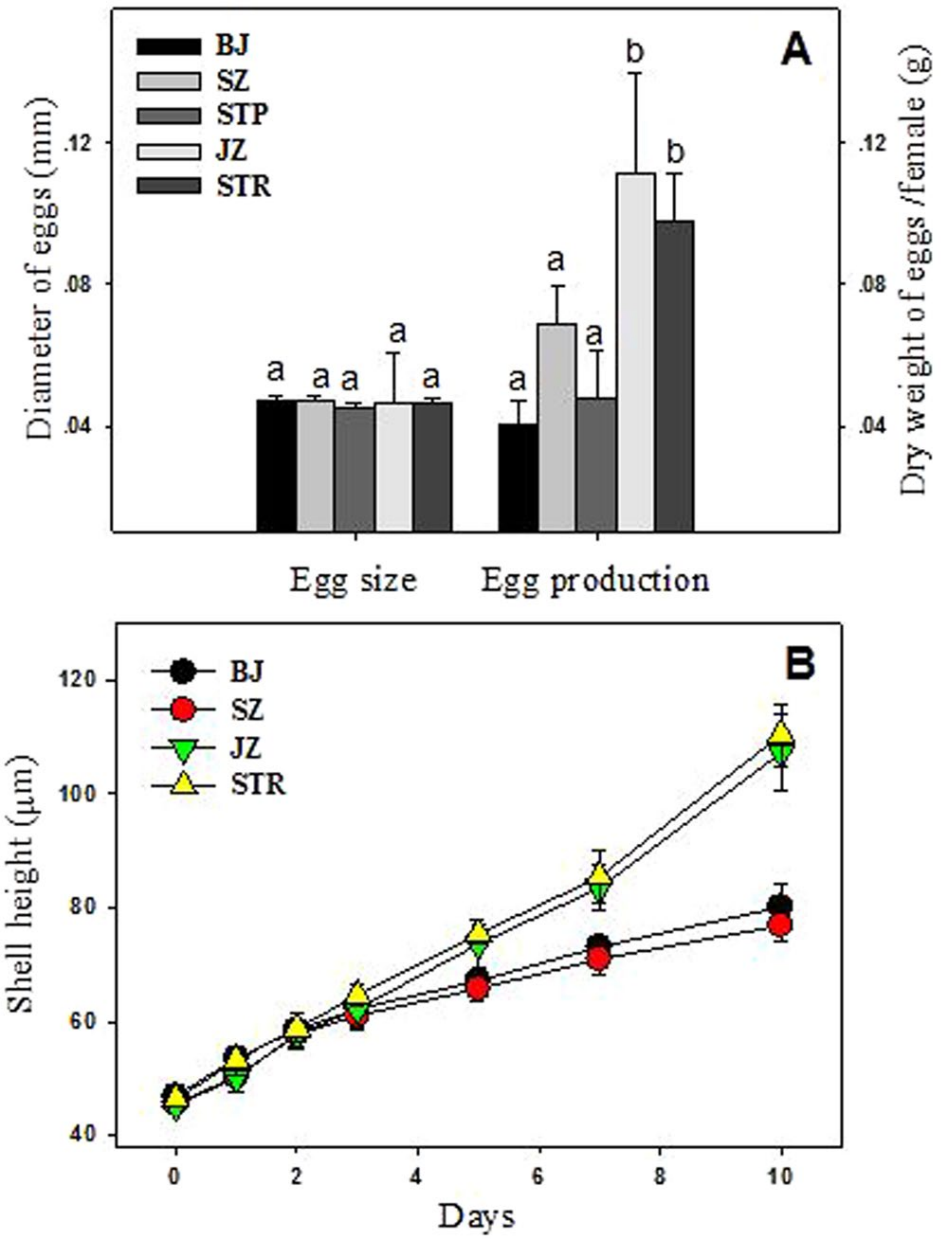
Egg production, egg size, and shell length of oyster larvae from each site. Values are given as mean ± SD, eggs from 8 females of BJ, SZ and STR site, and 7 females of JZ and STP site were used for the determination of egg production and egg size (the size of 200 oocyte were measured for each female), 60–100 larvae from at least 10 females and 4 males for each replicate were used for the measurement of shell height at each time point, two independent replicates for each site.

The egg production, egg size as well as shell height of larvae were determined to evaluate the potential impacts of long-term metal pollution on the reproduction and larval growth of oysters. Figure 2 shows no major difference in egg size among populations. However, the average egg production (g, dry weight per female) of oysters from the contaminated sites (BJ: 0.040, SZ: 0.069, STP: 0.048) was significantly lower than that of the relatively clean oysters (JZ: 0.111; STR: 0.098). Under natural conditions, the fecundity of bivalves is affected by many factors such as temperature, salinity, and food availability. As described in Table S4, the basic hydrological parameters including temperature, salinity and chlorophyll *a* content of different sampling sites were almost comparable, especially for BJ and JZ. The STR and STP site were located in the same Niutianyang estuary, and the distance between them was less than 2 kilometers, thus their environmental conditions were assumed to be comparable. Potential influence of organic pollutants has also been demonstrated in previous studies^41^. In the present study, the levels of organic contaminants in the oysters were not determined. According to previous reports, there was no noticeable contamination of organic contaminants in our sampling sites. A recent study demonstrated that the concentration of EDS (testosterone, oestrone and estradiol) in the sediment of metal contaminated site (close to BJ site in this study) of Jiulong River estuary was below detection limit (Ke and Wang, unpublished data). The level of PCBs and PAHs were relatively low in the sediment of the Jiulong River estuary, and the concentrations of these organic pollutants in oyster did not exceed the threshold of food criteria^42, 43^. Evidences also suggested that concentrations of organic pollutants (such as PAHs, PCBs, DDTs) in oysters of Pearl River estuary were generally at low levels^44–46^, and unlikely to result in obvious adverse impact on oyster populations. The contaminated sites (BJ, SZ and STP) of this study were considered to be the typical hotspots of trace metal pollution in estuary environments of Southern China. Therefore, we assumed that metal contamination primarily contributed to the reduced egg production of contaminated oyster populations.

**Figure 3:**
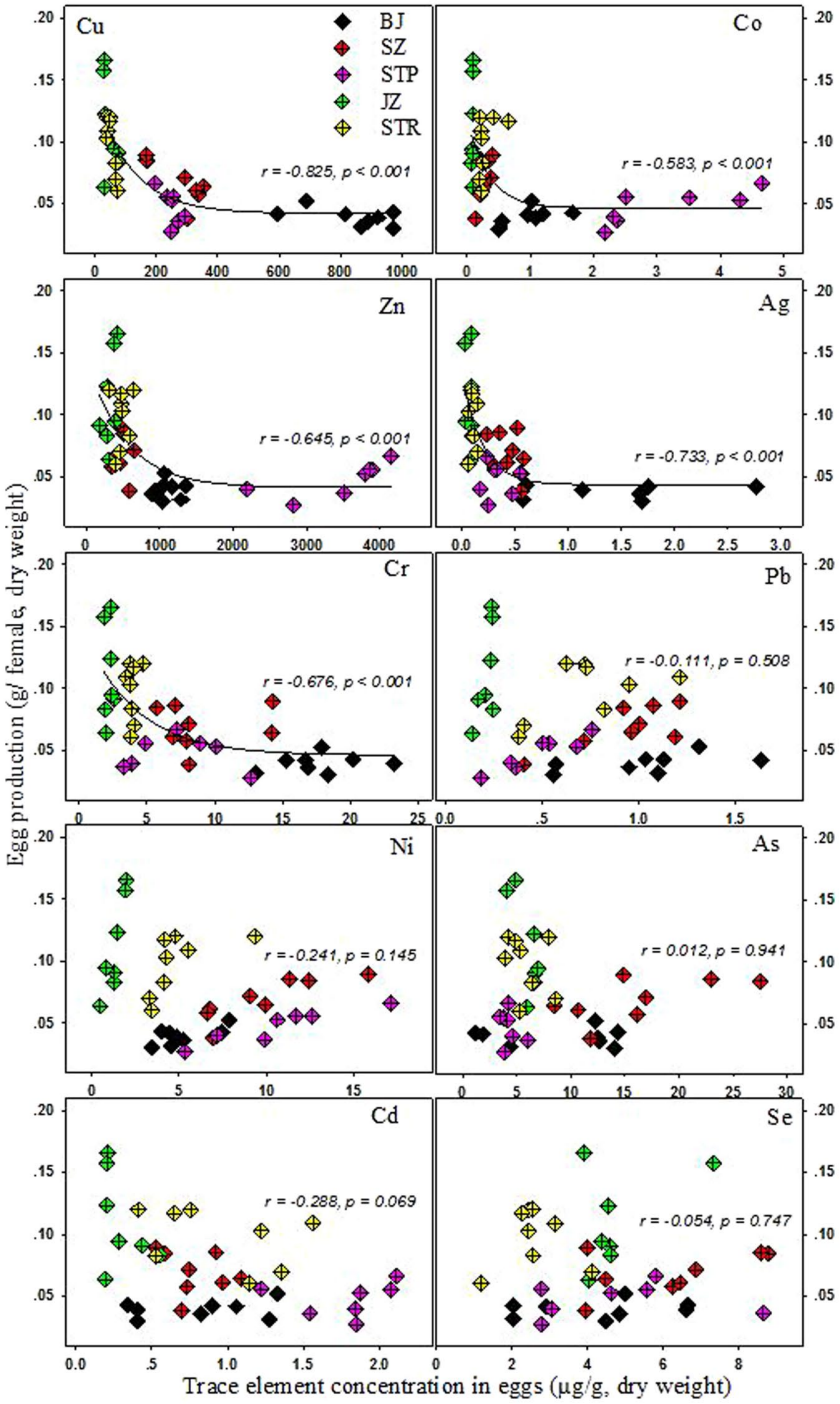
Relationship between trace element concentrations in eggs and egg production of females from all sampling sites. Each data point represents one female oyster (n = 8 for BJ, SZ and STR site, n = 7 for JZ and STP site). Correlation coefficient is Spearman rank correlation on untransformed data. The curves were non-linear regression generated with Sigma Plot 10.

To further explore the potential influences of metal contamination on reproduction, the correlation between trace element concentration in eggs and egg production of females was investigated in the present study. As described in Figure 3, egg production of females was significantly and negatively related to the accumulation of several metals (Cu, Zn, Co, Ag and Cr) in eggs, especially for Cu (r = −0.825, *p* < 0.001, Spear-man correlation) and Ag (r = −0.733, *p* < 0.001, Spear-man correlation), which provided direct evidence of the reduced fecundity of oyster populations associated with metal contamination in the field. The negative correlations between reproduction capacity and tissue concentration of Cu and Ag were also observed for bivalves such as *Potamocorbula amurensis* ^47^ and *Macoma balthica* ^48^ in San Francisco Bay.

Moreover, the lagged growth of larvae from contaminated sites was also observed in this study. Figure 2 shows that larvae growth of all sampling sites was comparable within the first 3 days, but the growth of larvae from contaminated sites (BJ and SZ) then became slower than that of the relatively clean oyster larvae (JZ and STR). Interestingly, the disparity between the 2 groups became more pronounced with time, indicating the adverse and latent effects of trace metal pollution on oyster offspring. The latent effects of many environmental factors such as nutritional deprivation^49^, food quality^50^ as well as acidification^51^ have been reported on the larvae of bivalves, but were firstly documented for metal exposure of adults in the field.

Several possible factors may be responsible for the reduced fecundity and lagged larval growth of contaminated oyster populations. First, maternal transferred metals may result in cell functional damage during the process of gametogenesis and early developmental stages, since metal accumulation in both eggs and larvae of contaminated oysters was much higher than that in eggs and larvae of the relatively clean oysters (Table 1). Of the elements investigated in our study, Cu, Zn and Ag need special attention. Oysters are hyper-accumulators of Cu and Zn. Although the transfer efficiency of these two metals was not high as described above (Table S2), the absolute concentrations of Cu and Zn in contaminated oyster eggs were up to 642 µg/g and 3456 µg/g dry weight, respectively. Evidence suggested that Cu and Zn in gonad as well as in eggs of contaminated oysters *Crassostrea gigas* mainly existed in soluble fraction other than in the membrane-bound granules (detoxified form)^52^, which may be harmful to gametogenesis. High percentages of degeneration, lysis, and resorption of oocytes as well as suppressed gametogenesis were also observed in metal contaminated (especially of Cu and Zn) oysters *Crassostrea angulata* ^53^. The reproductive toxicity of Ag was demonstrated in several aquatic organisms^54, 55^, and mechanism of Ag toxicity on reproduction included inhibition of vitellogenesis critical for oocyte development^56^. Brown *et al*.^47^ found that the reproductive activity of clam *Potamocorbula amurensis* in San Francisco Bay decreased by up to 30% when the annual mean tissue concentration of Ag was higher than 1 µg g^−1^ dry weight.

Cu, Zn and Ag are considered as the three most toxic elements to the early developmental stages of marine bivalves^57^. The concentrations of Cu, Zn and Ag in the newly hatched oyster larvae from contaminated sites were 11.0-fold, 4.4-fold and 7.8-folds higher than those from the relatively clean sites, indicating great risk to larval growth. A laboratory study demonstrated that the larval growth of *Crassostrea gigas* was significantly restrained when the larval body concentrations of Cd, Zn and Cu were up to 3-folds higher than those of the control larvae^58^. The Cu contents in oyster larvae from contaminated sites (BJ: 108.5 µg/g, SZ: 65.4 µg/g, dry weight) in our study were also significantly higher than the body median effect residues (49 µg/g dry weight) for the larvae of *Mytilus galloprovincialis* reported by Rosen *et al*.^59^. In future study, the critical residual concentrations of these metals based on eggs or larvae are needed to predict their toxicity on reproductive and offspring development of marine bivalves.

Second, the reduced reproductive output and larvae growth may reflect the change of energy allocation, where more resources were allocated to cope with metal stress such as detoxification and repairing damage. Several previous studies demonstrated that exposure to metals may impact the energy balance of bivalves attributing to increased maintenance cost, which finally led to a reduction in energy available for reproduction^60, 61^. The reduction of energy storage and nutrition materials in oocytes has been observed in bivalves due to metal stress^62^. Similarly, Ostrach *et al*.^2^ found that the contaminated fish larvae of striped bass presented a slower growth in comparison with the control larvae after 5 days of post-hatch, as a result of their less remaining yolk sac. Additionally, the rapid elimination of excessive maternally transferred metals in contaminated larvae during the early developmental stage could also consume more energy reserved for the later growth of oyster larvae.

Moreover, paternal effects may influence the growth and development of oyster offspring. The male oysters were also collected from the same contaminated sites in this study. As described in Table S4, the metal concentrations in male oysters were comparable to those of the corresponding metals in female oysters (Table 1), and also presented great variations among different oyster populations. Metts^63^ observed negative effects of paternal exposure of coal combustion waste on the reproductive success of southern toads (*Bufo terrestris*). However, the cell function damage, energy storage as well as paternal effects were not investigated in the present study. Further studies are needed to identify the relative importance of these factors.

To sum up, our study provided clear evidence of elevated accumulation of multiple trace elements in eggs and larvae of contaminated oyster *Crassostrea hongkongensis*. The excessively transferred metals in contaminated larvae were quickly eliminated during the early developmental stage. Metal efflux rates of contaminated oyster larvae were significantly higher than that of the larvae from relatively clean sites, which indicated the alteration of metal biokinetics in oyster offspring from contaminated enthronements. Moreover, contaminated oysters exhibited a lower fecundity as well as a slower larvae growth in comparison to the oysters sampled from the clean sites, indicating the potential adverse and latent effects of maternal metal transfer on the viability of oyster populations. Our findings may have significant implications for the environmental risk assessment and ecotoxicological research of bivalves in metal-contaminated estuaries.

## Methods

### Study sites and oyster sampling

Oysters of *Crassostrea hongkongensis* were sampled from four estuaries with contrasting pollution gradients of metals in South China. Specifically, two oyster populations were separately sampled from Jiulong River estuary (Baijiao, BJ site, 24°28′4″N, 117°56′22″E) and Jiuzhen estuary (Jiuzhen, JZ site, 24°02′15″N, 117°42′44″E) in Fujian Province, while the other three populations were sampled from Pearl River estuary (Shenzhen, SZ site, 22°36′06″N, 113°48′40″E) and Niutianyang estuary (Shantou: STP site, 23°38′20″N, 116°58′48″E, STR site, 23°37′08″N, 116°61′02″E) in Guangdong Province. Among the five sampling sites, BJ and SZ are the typical multi-metal contaminated sites, especially for Cu, Zn, Cr, Ni and Cd^7, 9^. The oyster population from STP was greatly contaminated with Zn, Cd and Ni^8^. The JZ site and STR site were relatively uncontaminated except the slight contamination by Zn, and were thus considered as the reference sites (Table 1). The basic hydrological parameters of each sampling sites are listed in Table S3.

Mature oysters of with similar size (11.7 ± 1.3 cm, Table S1) were collected from the oyster reefs during low tides in July 2013. For each site, 7–8 females and 5–6 males with ripe gonad were selected for the determination of trace element concentrations. In brief, oyster was dissected into gonad tissue and somatic tissue, respectively. Egg samples were obtained from the gonad tissue of females using a similar procedure as in fertilization experiment (described below). Diameters of eggs were determined simultaneously by microscopic measurements. In brief, a 50 µl gonad sample was mixed with 100 µl filtered clean seawater, and deposited on the glass slide. Egg pictures were taken using a camera (VIHENT, VIS500 CCD Color Video Camera) connected to a microscope (Olympus, CX-22LED); the diameters were measured using Image J software (n = 200 oocytes for each female). Samples of somatic tissue of oysters (both females and males) and eggs were store at −80 °C before trace element analysis. The remaining oysters (except STP site due to the limited sample size) from each site were conditioned separately in plastic tanks with 100 L clean seawater (pH 8.1, 20 psu) in the laboratory at 25 °C, and fed commercial clean algal power of *Spirulina* at a rate of approximately 1% of their soft tissue dry weight per day for the following experiments.

### Larvae growth assessment

Fertilization of oysters was carried out according to the previous study^21^, all the experimental seawater was collected 10 km offshore and free of metal contamination. Briefly, the gametes of oysters were obtained using the stripped method, and the oocytes and sperms of different oysters were observed after sieving through 100 µm for oocytes and 32 µm for spermatozoa under microscope, and the most reproductive gametes (regular oocytes and very mobile spermatozoa) were selected for the experiment. To obtain the next generation of different oyster populations, a minimum of 10 females and a minimum of 4 males were used for each replicate, and there were two independent experimental replicates for each site. Eggs from different females and spermatozoa from different males were pooled and mixed in separate containers. The mixed eggs were aged in filtered seawater (0.22 μm) for 30 min, and then the mixed sperm-dense suspension was added to the egg suspensions until approximately 10 sperms surrounded an egg as monitored by microscopy. Fifteen minutes after fertilization, the embryos were counted and cultured in plastic tanks with 30 L filtered seawater (pH 8.1, 25 psu) at 25 °C for 10 days. The embryo density was 60,000 l^−1^. After 48 h, larvae were fed *Isochrysis galbana* at a concentration of 10^5^ cells ml^−1^ once daily and all experimental solutions were renewed at 24-h intervals. Larval samples (about 100,000–120,000 larvae per replicate) were collected at 1, 2, 3, 5, 7 and 10 days, respectively. A fraction of the sampled larvae (about 100 per replicate) was fixed with 40% buffered formalin to measure the shell height. The remaining larvae samples and soft tissues of females were then stored at −80 °C for trace element analysis.

### Dynamic change of trace elements in larvae

Trace element concentrations in oyster larvae of different ages (1, 2, 3, 5, 7, 10 days) were determined to investigate the dynamic change of these elements during the early development stages. The dynamics of trace elements during the first two days was modeled by the following equation according to He and Wang^64^. For the newly hatched oyster larvae, most of the nutrient including trace elements was maternally derived. We assumed that the uptake of trace element in oyster larvae from seawater and food could be neglected during the first two days. Due to the rapid growth of oyster larvae, the potential effects of growth dilution were also included.

1 $${\rm{C}}={{\rm{C}}}_{0}{{\rm{e}}}^{-(ke+g)t};$$

2 $$W={W}_{0}{{\rm{e}}}^{{\rm{gt}}};$$

where C_0_ is the initial concentration of trace element in oyster larvae, *ke* is the efflux rate constant (d^−1^), t is time (d), g is the growth rate constant (g^−1^), *W*_*0*_ is the initial weight of oyster larvae (µg). The weight of oyster larvae was calculated with the relationship between body length (*L*, µm) and dry weight (*W*, µg): *W* = a*L* ^*b*^ according to Jespersen *et al*.^65^. The calculated value of a and b in this study, is 2.62 E^−8^ and 3.51, respectively.

### Trace element analysis

Samples of somatic tissues of oyster (females and males), eggs and larvae from each site were dried at 80 °C to obtain the dry weights, and then digested with nitric acid (70%, Merck Suprapur) and H_2_O_2_. For the analytic quality control, a standard reference material (SRM 1566b oyster tissues, National Institute of Standards and Technology, USA) was digested simultaneously. A total of 10 metals or metalloids (Ag, As, Cd, Co, Cr, Cu, Ni, Pb, Se, and Zn) were measured in this study. The concentrations of trace elements in all digested samples were determined by inductively coupled plasma-mass spectrometry (Agilent 7700x). The ICP-MS was calibrated with the external standards of multi-element standard solutions (Agilent), and appropriate internal standards (^45^Sc, ^72^Ge, ^118^In, ^209^Be) were selected to correct the instrumental drift and sensitivity change. A quality control sample (one of the diluted external standards) was repeatedly measured after each ten samples, and the relative standard deviations were less than 10%. Recoveries for SRM 1566b oyster tissues were 97–109% for Cu (measured value: 73.7 ± 4.21 µg/g, certified value of 71.6 µg/g), 91–98% for Zn (measured value: 1349 ± 55 µg/g, certified value of 1424 µg/g), 95–110% for Ni (measured value: 1.07 ± 0.08 µg/g, certified value of 1.04 µg/g), 91–102% for Co (measured value: 0.358 ± 0.022 µg/g, certified value of 0.371 µg/g), 90–98% for Cd (measured value: 2.33 ± 0.11 µg/g, certified value of 2.48 µg/g), 91–100% for Ag (measured value: 0.637 ± 0.032 µg/g, certified value of 0.666 µg/g) 91–101% for Pb (measured value: 0.296 ± 0.015 µg/g, certified value of 0.308 µg/g), 92–103% for As (measured value: 7.47 ± 0.42 µg/g, certified value of 7.65 µg/g). The recoveries of Cr and Se were not calculated as the certified concentrations are not available for them. The detection limit of ICP-MS was 0.012 μg/L for Cu, 0.024 μg/L for Zn, 0.016 μg/L for Cr, 0.019 μg/L for Ni, 0.006 μg/L for Pb, 0.008 μg/L for Cd, 0.003 μg/L for Co, 0.003 μg/L for Ag, 0.027 μg/L for As, and 0.491 μg/L for Se. The specific procedures of elemental analysis were according to the method described by Liu and Wang^66^. Reported results of element concentrations were not adjusted for recovery and expressed as dry tissue weight basis Trace metal clean technique was used during the whole experimental procedure.

### Ethic statement

All the methods were carried out in accordance with the approved guidelines. In addition, all the experimental protocols were approved by the Hong Kong University of Science and Technology safety committee (Hong Kong SAR, China).

### Statistics analysis

The concentrations of trace elements in females, males, eggs and larvae varied substantially among sites, these data as well as their related data such as egg burden proportions and efflux rate were thus log transformed before statistics analysis. All data related to the concentrations of trace elements (including efflux rate) and biological parameters (egg production, egg size, shell height, larvae growth rate) were compared between sites and elements using one-way ANOVA, Tukey post-hoc test. Prior to variance analysis, normality and homogeneity of data were confirmed by a Levene’s test. If the data with unequal variances, Kruskal-Wallis test was used for inter-group comparisons. Spear-man coefficients method was used for the correlation analysis. All statistical analyses were performed using SPSS 16.0 and *p* < 0.05 was accepted as significant. The plots were drawn in Sigma Plot 10.0.

## Electronic supplementary material

Supplementary information
