# Supplementary material for: Dynamics of maternally transferred trace elements in oyster larvae and latent growth effects
Source: Sci Rep. 2017 Jun 15;7:3580. doi: 10.1038/s41598-017-03753-2 (PMC5472574; doi:10.1038/s41598-017-03753-2)
Supplement: Supplementary file 1 — Supplementary information [file 41598_2017_3753_MOESM1_ESM.doc]

**Supporting Information**

**Dynamics of maternally transferred trace elements in oyster larvae and latent growth effects**

Nanyan Weng^1, 2^, Wen-Xiong Wang^1, 2^∗

*^1^ Marine Environmental Laboratory, HKUST Shenzhen Research Institute, Shenzhen 518057, China*

*^2^ Center for Marine Environmental Chemistry and Toxicology (CMECT), College of Environment and Ecology, Xiamen University, Xiamen 361005, China*

*Corresponding author, email: [wwang@ust.hk](mailto:wwang@ust.hk)

(Four tables and one figure)

Table S1. The shell size of oysters sampled from each site. Data are mean±SD. N is the number of oysters used for the measurement.

| Site | BJ | SZ | JZ | STR | STP |
| --- | --- | --- | --- | --- | --- |
| Shell length (cm) | 11.5±1.3 | 12.0±0.8 | 11.8±1.5 | 12.5±1.41 | 10.9±1.6 |
| N | 14 | 14 | 14 | 14 | 14 |

Table S2. The ratio of egg burdens of trace element to the total body burdens of corresponding element in female oysters from each site. Values are given as mean±SD (n = 8 for BJ, SZ and STR site; n = 7 for JZ and STP site).

| Egg burden/ total burden (%) | | | | | |
| --- | --- | --- | --- | --- | --- |
|  | JZ | STR | SZ | BJ | STP |
| Cu | ^C^2.35±0.22c | ^C^2.45±0.53b | ^B^1.02±0.36a | ^A^0.75±0.10a | ^B^1.14±0.18a |
| Zn | ^A^0.95±0.09a | ^A^0.92±0.19a | ^A^0.71±0.25a | ^A^0.82±0.11a | ^A^0.86±0.13a |
| Cr | ^A^31.7±3.50f | ^C^77.4±16.8f | ^B^67.1±23.9e | ^A^27.6±3.73e | ^B^56.2±8.83e |
| Ni | ^B^14.3±1.54e | ^C^28.4±6.61e | ^C^21.7±4.73d | ^A^10.9±1.48d | ^B^15.7±2.46d |
| Co | ^B^8.09±0.56d | ^B^10.8±2.34c | ^B^6.58±2.34c | ^A^3.99±0.54b | ^B^8.75±1.38c |
| Cd | ^C^1.68±0.12b | ^C^1.47±0.32a | ^A^0.58±0.21a | ^A^0.63±0.08a | ^B^0.93±0.15a |
| Ag | ^A^2.71±0.30c | ^A^2.34±0.51b | ^A^2.00±0.71b | ^A^2.19±0.30b | ^A^2.95±0.46b |
| Pb | ^B^6.01±1.28d | ^C^15.3±3.31d | ^C^10.1±3.60c | ^C^11.2±1.52d | ^A^3.33±0.52b |
| Se | ^B^16.4±2.08e | ^A^9.68±2.10c | ^A^12.9±4.58c | ^A^9.17±1.24d | ^A^10.2±1.61c |
| As | ^B^14.9±1.56e | ^B^18.9±4.10d | ^C^28.6±4.17d | ^A^5.94±0.80c | ^B^14.0±2.20cd |

Different letters indicate significant difference among elements and sites at *p* < 0.05 level (one-way ANOVA, Turkey test), lowercase letters are used for different elements, and capital letters are used for different sites.

Table S3. The values (means or ranges) of general hydrological parameters of each sampling site. Temp-temperature, Sal-salinity, DO-dissolved oxygen, Chl *a-*chlorophyll *a*; n.d.- not determined.

|  | Temp (℃) | Sal (‰) | DO (mg/l) | pH | Chl *a* (µg/l) |
| --- | --- | --- | --- | --- | --- |
| BJ | 22.6^a^  (14.0-30.2) | 21.0^a^  (14.6-25.0) | 7.79^a^  (6.60-8.77) | 7.76^a^  (7.36-7.96) | 6.48^a^  (0.96-32.97) |
| JZ | 22.6^a^  (14.2-30.6) | 15.8^a^  (11.4-23.1) | 7.61^a^  (6.84-8.83) | 7.78^a^  (7.42-7.97) | 8.51^a^  (1.05-37.66) |
| SZ | 16.6-30.3^b^ | 2.1-19.4^b^ | 7.43-8.10^b^ | 7.75-7.97^b^ | 4.61^c^  (2.22-8.00) |
| STR,STP | 27.5±0.4^d^ | 10.0±1.3^d^ | 7.21±0.19^d^ | n.d. | 17.01±0.93^d^ |

1. Cited from Weng and Wang^1^, the data are given as annual mean and range;
2. Monitoring results during two consecutive years of SZ sites (unpublished data), the data are given as range;
3. Cited from Zhou et al.^2^, the data is given as mean and range of Pearl River Estuary;

Cited from Li et al.^3^, the data are given as mean±SD of the Niutianyang estuary in summer season.

Table S4. Trace element concentrations in male oysters from each site. Values are given as mean ± SD (n = 6 for BJ, SZ and STR site; n = 5 for JZ and STP site).

| Metal concentrations (µg/g dry weight) | | | | | |
| --- | --- | --- | --- | --- | --- |
|  | BJ | SZ | JZ | STR | STP |
| Cu | 11181±2186c | 4313±1474b | 426±71.5a | 395±146a | 3008±682b |
| Zn | 16671±1159b | 9753±1785ab | 7804±1937a | 12136±3569ab | 55673±9423c |
| Cr | 4.19±0.73b | 0.76±0.28a | 0.38±0.11a | 0.50±0.13a | 0.88±0.13a |
| Ni | 5.71±0.84b | 6.11±1.75b | 2.03±0.56a | 2.62±0.47a | 9.56±2.71c |
| Co | 2.75±1.21b | 0.59±0.25a | 0.30±0.07a | 0.63±0.23a | 6.54±1.26c |
| Cd | 18.5±4.08bc | 22.2±3.01cd | 3.92±0.56a | 12.8±3.90b | 27.5±5.79d |
| Ag | 7.87±2.92c | 3.32±1.41b | 0.51±0.12a | 0.86±0.26ab | 1.60±0.74ab |
| Pb | 1.62±0.37b | 1.35±0.32b | 0.75±0.15a | 0.83±0.15a | 0.85±0.12a |
| Se | 5.69±0.35b | 6.97±0.64c | 5.89±40.48b | 4.67±0.59a | 5.73±0.43b |
| As | 19.0±1.99d | 6.96±0.52bc | 8.33±1.59c | 5.14±1.11ab | 3.58±0.27a |

Different letters in the same row indicated significant difference among sites at *p* < 0.05 level (one-way ANOVA, Tukey test)

Figure S1 Relationship of trace element concentrations between females and their newly hatched larvae from each site. Each data point represents one independent replicate (two replicates for each site, about 100,000-120,000 larvae from at least 10 females and 4 males for each replicate). Correlation coefficient is Spearman rank correlation on untransformed data. The curves were linear regression generated with Sigma Plot 10.

**References**

1. Weng, N.Y. & Wang, W.-X. Variations of trace metals in two estuarine environments with contrasting pollution histories. *Sci. Total Environ.* **485–486,** 604–614 (2014).
2. Zhou, W., *et al.* Bacterioplankton dynamics along the gradient from highly eutrophic Pearl River Estuary to oligotrophic northern South China Sea in wet season: implication for anthropogenic inputs. *Mar. Pollut. Bull.* **62**, 726-733 (2011).
3. Li, C. B., *et al.* Survey of cyanomyovirus abundance in Shantou coastal water by g20. *Chin. J. Oceanol. Limnol.* **33,** 604-615 (2015).
